# Leptin in Children with Newly Diagnosed Type 1 Diabetes: Effect of Insulin Therapy

**DOI:** 10.1155/EDR.2001.121

**Published:** 2001

**Authors:** Kenneth L. McCormick, Gail J. Mick, Lisa Butterfield, Hugh Ross, Elaine Parton, Joan Totka

**Affiliations:** 1 University of Alabama Children's Hospital ACC 608 1600 Seventh Ave. South Birmingham Alabama 35233-0011 USA; 2 Medical College of Wisconsin Milwaukee WI 53226 USA

## Abstract

Leptin, the gene product of adipose tissue that signals caloric plentitude *via* central nervous system receptors, may also have diverse peripheral metabolic actions. Of paramount interest has been the potential interaction(s) between leptin and insulin. Insofar as insulin alters leptin secretion/action (or *vice versa*), dysregulation of this system could contribute to disease states such as diabetes.

The purpose of this study was to examine the effect of exogenous insulin on serum leptin in children with newly-diagnosed Type 1 diabetes. Since these patients are hypoinsulinemic (insulindeplet. ed) at diagnosis, they present an ideal opportunity to examine the effect of insulin repletion on serum leptin. Seventeen patients were enrolled. At baseline (prior to insulin therapy), leptin levels were 4.3 ± 1.1ng/ml; they were not statistically related to the baseline serum insulin or illness severity. There was no significant change in serum leptin before, shortly (1–6 days) or several weeks (3–26 weeks) after insulin treatment even when the data was corrected for changes in BMI, hemoglobin A_1C_, and daily insulin dose. Since repletion of the insulin deficiency that is present in non-acidotic, ambulatory patients with new onset Type 1 diabetes did not alter serum leptin, these results argue against an effect of insulin on serum leptin in the absence of the acute diabetic ketoacidosis. Because as the recuperative months following the diagnosis of new onset Type 1 diabetes are marked by weight gain, the absence of a rise in serum leptin might also indicate either an adaptive (weight permissive) or pathologic (impaired secretory) deficit.


*Int. Jnl. Experimental Diab. Res.*, Vol. 2, pp. 121–127
Reprints available directly from the publisher
Photocopying permitted by license only

© 2001 OPA (Overseas Publishers Association) N.V.
Published by license under
the Harwood Academic Publishers imprint,
part of Gordon and Breach Publishing,
a member of the Taylor & Francis Group.
Printed in the U.S.A.

### Leptin in Children with Newly Diagnosed Type 1 Diabetes: Effect of Insulin Therapy

KENNETH L. McCORMICK[a,*], GAIL J. MICK[a], LISA BUTTERFIELD[b], HUGH ROSS[b], ELAINE PARTON[b] and JOAN TOTKA[b]

[a]*University of Alabama, Birmingham, Alabama 35233;* [b]*Medical College of Wisconsin, Milwaukee, WI, 53226*

*(Received 13 December 2000; In final form 28 February 2001)*

**Leptin, the gene product of adipose tissue that signals caloric plentitude *via* central nervous system receptors, may also have diverse peripheral metabolic actions. Of paramount interest has been the potential interaction(s) between leptin and insulin. Insofar as insulin alters leptin secretion/action (or *vice versa*), dysregulation of this system could contribute to disease states such as diabetes.**

**The purpose of this study was to examine the effect of exogenous insulin on serum leptin in children with newly-diagnosed Type 1 diabetes. Since these patients are hypoinsulinemic (insulin-depleted) at diagnosis, they present an ideal opportunity to examine the effect of insulin repletion on serum leptin. Seventeen patients were enrolled. At baseline (prior to insulin therapy), leptin levels were 4.3 ± 1.1 ng/ml; they were not statistically related to the baseline serum insulin or illness severity. There was no significant change in serum leptin before, shortly (1–6 days) or several weeks (3–26 weeks) after insulin treatment even when the data was corrected for changes in BMI, hemoglobin $A_{1C}$, and daily insulin dose. Since repletion of the insulin deficiency that is present in non-acidotic, ambulatory patients with new onset Type 1 diabetes did not alter serum leptin, these results argue against an effect of insulin on serum leptin in the absence of the acute diabetic ketoacidosis. Because as the recuperative months following the diagnosis of new onset Type 1 diabetes are marked by weight gain, the absence of a rise in serum leptin might also indicate either an adaptive (weight permissive) or pathologic (impaired secretory) deficit.**

*Keywords*: Leptin; Insulin; Type 1 diabetes

### INTRODUCTION

Leptin, the product of the obese (ob) gene, is a 16 kd peptide which is produced primarily by adipose tissue[1] although it is now evident that the gene is also transcribed in stomach and placenta.[2] Although leptin's precise role in humans is unsettled, in rodents the hormone controls body weight and energy expenditure through feedback signaling *via* hypothalamic receptors.[3] In all normal mammals studied to date, the plasma concentration of leptin strongly correlates with percent body fat.[4] Additionally, in humans, there is a gender effect in so far as females tend to have higher plasma levels even after adjusting for body fat.[5,6] The

*Address for correspondence: University of Alabama, Children's Hospital, ACC 608, 1600 Seventh Ave. South, Birmingham, AL 35233-0011. Tel.: 205-939-5260, Fax: 205-939-9821, e-mail: kmccormick@peds.uab.edu predominant hormones which are felt to govern leptin ob mRNA and plasma concentrations, both *in vivo* and *in vitro*, are glucocorticoids and insulin.[7-10,11] Of these two, from a teleological standpoint, insulin is the choice of most to be the preeminent physiological regulator and would account for the enhanced production of leptin in adipocytes which accompanies an increase in caloric intake. A correlation between serum leptin and insulin can be found,[12] even after normalizing the data for percent body fat. Previous studies have found that either acute insulin treatment in lean rats or food ingestion increases ob gene expression and/or serum leptin levels.[13] In humans, a 1996 study found that supra physiologic hyperinsulinemia increases leptin levels after only 4 hours.[14] However, antithetically, several reports have not been able to show that acute exposure to insulin regulates leptin levels. For instance, in two separate studies in humans, no effect of exogenous insulin on serum leptin levels was observed unless hyperinsulinemia persisted for 24 hours or more.[15,16] In fact, we[17] and others[18-20] have evidence that in the presence of steroids, insulin actually attenuates leptin secretion in either cultured adipocytes or adipose tissue.

To further define the relationship between serum leptin and insulin dynamics, we studied children with new-onset Type 1 diabetes who were managed in an outpatient care program. They provide a prime opportunity to examine the impact of exogenous insulin on circulating leptin levels in humans with profound hypoinsulinism. Our study is unique in that the children studied were largely nonketotic as determined by serum bicarbonate (only 3 of 17 were admitted for intravenous treatment of mild ketoacidosis). Since nearly all Type 1 diabetics demonstrate significant weight gain after insulin treatment,[21] this fact alone, regardless of the aforementioned direct effects of insulin per se on leptin synthesis, should lead to an increase in serum leptin. We prospectively followed serum leptin concentrations in 17 untreated new onset diabetics from baseline (prior to insulin therapy) through two successive follow-up clinic visits.

### METHODS

### Subjects

Informed consent was obtained from the parents and patients prior to enrollment. The study was approved by the Institutional Review Board. All untreated Type 1 new-onset diabetic children referred to the endocrine unit were asked to participate. Patients were excluded from the study for the following reasons: abnormal thyroid function or antibodies, previous history of chronic disease, prior insulin therapy, history of any concurrent drug treatment or the development of an intercurrent illness during the study period. The study group comprised 17 untreated Type 1 diabetics (7 males/10 females, mean age 8.6 yrs $\pm$ 4.9 (range 2.6–17.2), mean blood glucose (mM) 23.1 $\pm$ 11.2 (range 13.4–53.1)). Only 3 required brief hospitalization for intravenous hydration and mild ketoacidosis, defined as a serum total $CO_2$ below 17 mM.

Parenteral fluids (3 of 17 patients), if necessary, and insulin requirements and adjustments were under the supervision of the endocrinology service. Upon recruitment (baseline), detailed information was obtained regarding duration of symptoms, weight loss (if available), history of vomiting, or history of anorexia or polyphagia for the 24 hrs preceding insulin treatment. Heights and weights were obtained at baseline and at both subsequent follow-up visits. The first follow-up visit was shortly after diagnosis (range 1–6 days) and the other several weeks thereafter (range = 2.9–25.9 weeks; mean 18.2 $\pm$ 6.0 SD). At the latter appointment, the mean daily dose of insulin per kg body weight was recorded.

### Assays

As to blood studies, at baseline (prior to insulin treatment) the following were measured: venous pH, electrolytes, total $CO_2$,

BUN, creatinine, glucose, insulin, hemoglobin $A_1c(HbA_1c)$, and, of course, leptin. Blood leptin and glucose were analyzed at both follow-up visits (herein referred to as the first and second return visits) while $HbA_{1C}$ was measured a baseline and the second follow-up visit.

Serum leptin concentrations were measured in triplicate by radioimmunoassay (RIA) using a commercially available kit (Linco Research, St. Charles, MO) with a limit of sensitivity of 0.5 ng/ml. The intra assay coefficient of variation was 7.1% at 10 ng/ml and the respective between-assay coefficient was 6.8%. During the course of the study each subject had 3 separate leptin measurements (baseline plus 2 follow-up visits) and these were batched together in the same assay. Prior to measurment, sera was stored at −70°C. $HbA_{1C}$ was measured by immunoassay with a nondiabetic range 4.2–6.2% (DCA 2000TM Analyzer, Miles Inc., Tarrytown, NY). Immunoreactive human insulin was measured by a commercially available assay (Diagnostic Products Corp., Los Angeles, CA). At 15 μU/ml insulin the respective intra- and interassay coefficients of variation were 6.9% and 8.9%. Routine chemistries (venous pH, electrolytes, total $CO_2$, BUN, creatinine) were obtained through the hospital clinical lab.

### Statistics

Statistical comparisons were done by Students t-test and regression analysis. The potential effect of variables such as the clinical severity at presentation, insulin dose, the percent change in $HbA_{1C}$ and the % change in BMI were tested using the analysis of variance (ANOVA). All values are expressed as the mean ± SD.

### RESULTS

### Leptin and Insulin Levels at Baseline

The mean serum leptin level, insulin, $HbA_{1C}$, and body mass indices are shown in Table I. There was no statistical relation between the baseline serum insulin and leptin concentrations. Moreover, baseline leptin levels were not statistically related to the presence or severity diabetes symptoms at presentation (duration of symptoms, history of vomiting, % weight loss, or history of either polyphagia or anorexia).

TABLE I Patient characteristics and blood values at the baseline and final follow-up visits

| | Baseline | Final visit |
|---|---|---|
| Body mass index | 15.5 ± 1.8 | 16.9 ± 2.0 |
| Weight (kg) | 26.8 ± 11.8 | 30 ± 11.0 |
| Leptin (ng/ml) | 4.3 ± 1.1 | 5.1 ± 3.5 |
| $HbA_{1C}$ | 10.9 ± 1.6 | 8.6 ± 1.1 |
| Insulin (μU/ml) | 3.7 ± 3.6 | – |

Data are presented as mean ± SD on the 17 enrolled patients at baseline (before insulin therapy) and at their final (or second return) visit as per methods.

### Serum Leptin: Response to Insulin Therapy

The temporal changes in serum leptin concentration in each of the 17 patients are shown in Figure 1. The mean serum leptin level (ng/ml) at the first and second return visits were 4.2 ± 1.0 and 5.1 ± 3.3 ng/ml. These follow-up leptin concentrations were not statistically different from the basal leptin. The serum leptin levels of the three children who presented with mild ketoacidosis and, therefore, received intravenous fluids and insulin, were examined separately to determine if leptin levels might change during the course of DKA therapy in these patients. The temporal change in serum leptin was not significant: the mean concentrations were 4.7 ± 1.0 (at baseline), 4.4 ± 1.0 (at 0.3–0.4 weeks) and 5.3 ± 3.1 ng/ml (at 9.5–26 weeks).

All of the patients gained weight after insulin treatment (Tab. I). There was no correlation ($p > .05$) between the % changes in BMI (as measured at the 2nd return visit) and the % changes in serum leptin (Fig. 2). With the exception of 2 patients, the diabetic

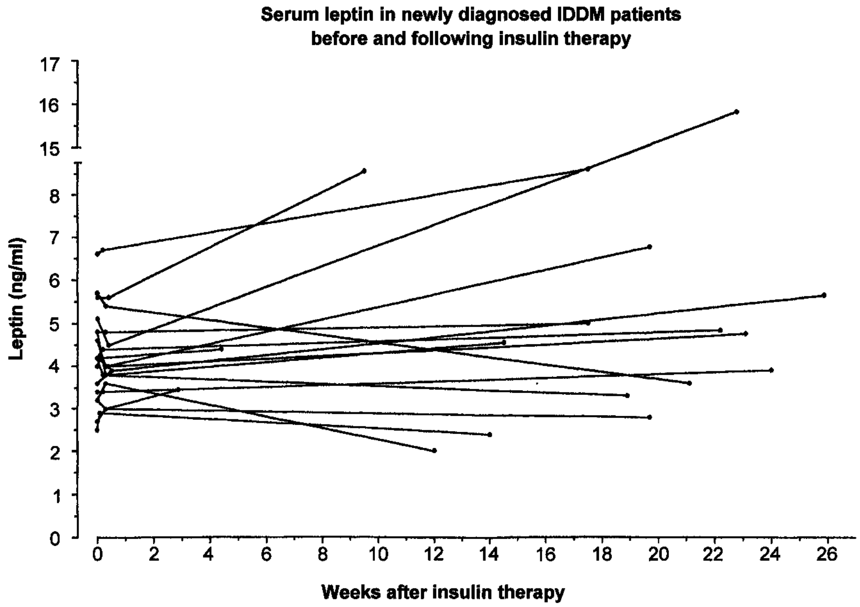

FIGURE 1 Temporal change in serum leptin. The temporal changes in serum leptin for the 17 study patients are shown. Blood samples were obtained and measured per methods.

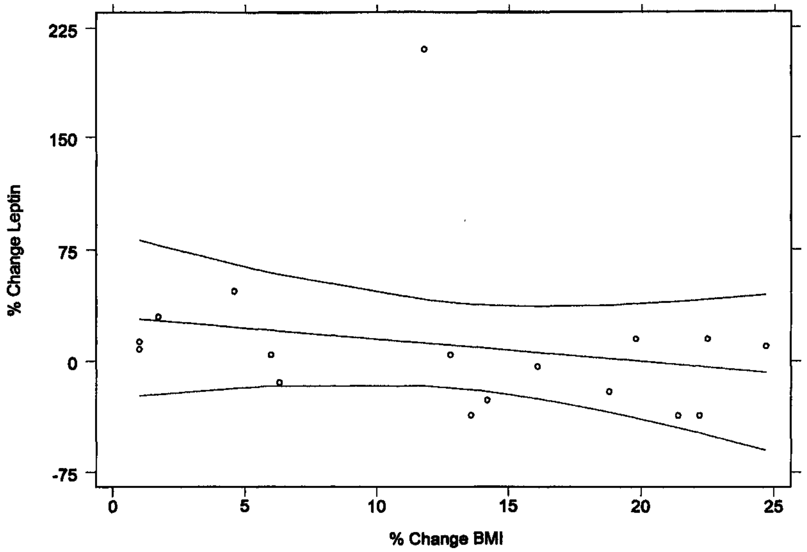

FIGURE 2 Comparison between the percent changes in serum leptin and body mass index (BMI) in children with newly IDDM during their initial three months of insulin therapy. Correlation between the percent change in serum leptin and the percent change in BMI in the 17 study patients. Data was analyzed by linear regression; no statistically significant relationship was evident.

children had a reduction in $HbA_{1C}$ values between baseline and the final examination (Tab. I). Nevertheless, when the individual percent changes in $HbA_{1C}$ were plotted against the percent change in serum leptin, there was no significant correlation (data not included). Finally, the patient's daily dose of insulin per kg body weight at the final visit did not correlate with their percent change in serum leptin (not shown).

### DISCUSSION

The primary goal of this study was to determine whether serum leptin would change over the first few months of insulin replacement therapy in children with newly diagnosed Type 1 diabetes. The study population was composed of 17 patients 14 of which were entirely managed as outpatients in an clinic program for newly diagnosed patients. We reasoned that if leptin is regulated by insulin, then serum leptin should change (increase) during the transition from the hypoinsulinemia that characterizes new onset Type 1 diabetes at presentation to full insulin replacement over the ensuing months. Moreover, since weight gain is usual during this same period, that in itself should increase leptin. Contrary to our hypothesis, we found no significant change in leptin despite resolution of hyperglycemia, increased weight and statistical correction for potential confounding variables.

Studies examining the direct effects of insulin on blood leptin levels have largely supported a positive correlation between the two hormones. The majority of *in vivo* studies have shown (both in clinically well, non-diabetic individuals and patients with either Type 1 or Type 2 diabetes) that 3–6 hours of euglycemic hyperinsulinemia increases serum leptin by approximately 50%.[14,22–25] Analogously, serum leptin is elevated in patients with insulinoma but normalizes after tumor resection.[26] Discordant with this, two studies found no change in serum leptin after 5 hours of euglycemic hyperinsulinemia in either normal or diabetic subjects.[12,15]

In adult nonobese men with well-controlled Type 1 diabetes, fasting serum leptin levels were over twice non-diabetic levels but did not correlate with fasting insulin levels. In this same study, four hours of euglycemic hyperinsulinemia increased serum leptin by 25% in controls but not in the subjects with diabetes.[22] Healthy, lean prepubertal children with stable Type 1 also have higher fasting serum leptin levels relative to controls, an association which could be due to insulin therapy.[27]

A molecular mechanism has been proposed to explain how insulin and nutritional signals might regulate serum leptin. The candidate transcription factor, adipocyte determination differentiation dependent factor 1/sterol regulatory element binding protein 1 (ADD1/SREBP1)I which is activated by insulin and prior feeding causes increased leptin secretion and ob mRNA in fat tissue.[26] Despite this, however, *in vitro* studies of cultured fat tissue and cells have yielded opposing results concerning the sustained effect of insulin on leptin secretion.[18,19,28,29]

Several recent studies have addressed the effect of acute insulin therapy in insulin deficient diabetes (either newly diagnosed patients with Type 1 diabetes in ketoacidosis or ketosis[30,31] or insulin withdrawal.[32] In a study of 19 newly diagnosed children with Type 1 diabetes who presented in ketosis and/or ketoacidosis leptin levels were 46% reduced (compared to controls) just prior to insulin treatment.[30] These depressed leptin levels corrected to 66 and 77% of normal after 1 and 3–5 days, respectively, commensurate with intensive (every 4–6 hours) insulin administration for the first 24 hours of therapy followed by conventional twice daily insulin thereafter. Our results differ in that we detected no change in serum leptin over a similar observation period. These contrasting results could be due to differences in the degree of illness severity between patient cohorts. Upon entrance into the aforementioned study,[30] 15/19 of the patients were in ketosis and/or ketoacidosis, however, specific acid–base measurements were not provided. In contrast, only 3/17 of our patients were in mild ketoacidosis as defined by a serum bicarbonate < 17 mM. We did not enroll a control group since the purpose of this study was to prospectively follow the change in serum leptin during the transition from a state of hypoinsulinemia to one of insulin repletion. With this study design, patients served as their own controls. Evidence corroborating the positive change in insulin status in this cohort was the increase in BMI (all patients) and reduction in $HbA_{1C}$ (all but 2 patients). Furthermore, it is well recognized that new-onset patients with Type 1 diabetes and classic symptoms (polydipsia, polyuria) are hypoinsulinemic at diagnosis and that conventional insulin therapy exposes tissues to supraphysiologic concentrations of insulin.[33] In another study of sick children with diabetic ketoacidosis [31] serum leptin were also about 50% decreased at presentation and normalized rapidly with 24 hours of intensive insulin and fluid therapy. Of interest, in a subset of these patients who were well enough to eat, there was a less dramatic rise in leptin over the study period. It would be of interest to know if these patients were less acidotic on presentation. This study differs from ours in that it exclusively examines the first 24 hours of treatment of acute diabetic ketoacidosis. We avoid the potential biochemical, hormonal and fluid and electrolyte variables associated with ketoacidosis by studying clinically well patients and following them for several months.

Several observations are noteworthy regarding the potential impact of the insulin deficient state on serum leptin. Firstly, while the observed changes in serum leptin that occur during the initial insulin therapy in patients with new onset diabetes could be due to the insulin per se, the correction of other concomitant abnormalities in the metabolic and hormonal milieu might also affect serum leptin. Along these lines, insulin deficiency might be associated with changes in serum protein binding and/or the binding of leptin in peripheral pools with subsequent enhancement of leptin clearance.[34-36] The importance of leptin clearance over leptin production in determining ambient serum leptin has been proven.[35,37,38] Moreover, even free fatty acids, which increase with insulin deficiency, bind to leptin and could potentially effect leptin clearance.[39] Catecholamines, which suppress leptin, might also have a role in lowering leptin levels in new diagnosed Type 1 diabetic patients. In summary, since the metabolic/hormonal milieu may vary with illness severity, these attendant factors could, in part, explain the disparate results (both in human and rodent data) regarding the action of insulin repletion on serum leptin in insulin-deficient diabetes.

Our study is unique in that it examines a cohort of children with mild non-acidotic, new onset Type 1 diabetes. Given that the patients were not direly ill, the impact of hypoinsulinemia and subsequent insulin repletion on serum leptin could be prospectively examined independent of ketoacidosis (only three patients had serum bicarbonate less than 17 mM). This is corroborated by data demonstrating the absence of a statistical relationship between either the baseline leptin and the percent change in leptin, baseline insulin levels, or changes in weight, BMI or $HbA_{1C}$. Also, the absence of an effect of insulin replacement on leptin levels in our patients cannot be due to inadequate insulin repletion since all patients had a rapid resolution of symptomatic hyperglycemia and all had an increase in BMI and reduction $HbA_{1C}$ (15/17 patients). Notwithstanding the increase in body weight over the observation period, there was still no significant change in serum leptin despite up to 3 months of insulin replacement. Our results demonstrate that in the non-ketotic, insulin-deficient state of clinically stable new onset Type 1 diabetes, insulin replacement therapy does not affect serum leptin despite improved glycemic control and weight gain. Given that our data did not show increased leptin with weight gain a possible corollary to these observations may be that patients with Type 1 diabetes have a relative deficiency (adaptive or disease-related) in leptin secretion in this initial post diagnosis time period.

### *Acknowledgements*

Research funded by Max McGee Juvenile Diabetes Research Fund, Medical College of Wisconsin.

### *References*

[1] Zhang, Y. *et al.* (1994). Positional cloning of the mouse obese gene and its human homologue. *Nature*, **372**, 425–432.

[2] Bado, A. *et al.* (1998). The stomach is a source of leptin. *Nature*, **394**, 790–793.

[3] Campfield, L. A. *et al.* (1995). Recombinant mouse ob protein: evidence for a peripheral signal linking adiposity and central neural networks. *Science*, **269**, 546–549.

[4] Maffei, M. *et al.* (1995). Leptin levels in human and rodent: measurement of plasma leptin and ob/RNA in obese and weight-reduced subjects. *Nature Med.*, **1**, 1155–1161.

[5] Considine, R. V. *et al.* (1995). Serum immunoreactive-leptin concentrations in normal-weight and obese humans. *N. Engl. J. Med.*, **334**, 292–295.
[6] Ostlund, R. E. *et al.* (1996). Relation between plasma leptin concentration and body fat, gender, diet, age, and metabolic covariates. *J. Clin. Endocrinol. Metab.*, **81**, 3909–3913.
[7] Saladin, R. *et al.* (1995). Tranisent increase in obese gene expression after food or insulin administration. *Nature*, **377**, 527–532.
[8] Slieker, L. *et al.* (1996). Regulation of expression of ob mRNA and protein by glucocorticoids and cAMP. *J. Biol. Chem.*, **271**, 5301–5304.
[9] Wabitsch, M. *et al.* (1996). Insulin and cortisol promote leptin production in cultured human fat cells. *Diabetes*, **45**, 1435–1438.
[10] Mantzoros, C. and Moschos, S. (1998). Leptin: in search of role(s) in human physiology and pathophysiology. *Clin. Endocrinol.*, **49**, 551–567.
[11] Dagogo-Jack, S. (1999). Regulation and possible significance of leptin in humans: leptin in health and disease. *Diab. Rev.*, **7**, 23–38.
[12] Dagogo-Jack, S. *et al.* (1996). Plasma leptin and insulin relationships in obese and nonobese humans. *Diabetes*, **45**, 695–698.
[13] Koopsmans, S. J. *et al.* (1998). Effect of hyperinsulinemia on plasma leptin concentrations and food intake in rats. *Am. J. Physiol.*, **274**, E998–E1001.
[14] Utriainen, T. *et al.* (1996). Supraphysiological hyperinsulinemia increases plasma leptin concentrations after 4 h in normal subjects. *Diabetes*, **45**, 1364–1366.
[15] Kolaczynski, J. W. *et al.* (1996). Acute and chronic effect of insulin on leptin production in humans. Studies *in vivo* and *in vitro*. *Diabetes*, **45**, 699–701.
[16] Boden, G. *et al.* (1997). Effects of prolonged hyperinsulinemia on serum leptin in normal human subjects. *J. Clin. Invest.*, **100**, 1107–1113.
[17] Mick, G., Fu, C.-L. and McCormick, K. L. (1998). Effect of insulin, metformin and glybenclamide on leptin secretion from rat adipose tissue. *Diabetes* (abstract), **47**(Suppl. 1), p. A408.
[18] Reul, B. A. *et al.* (1997). Insulin and insulin-like growth factor 1 antagonize the stimulation of ob gene expression by dexam-ethasone in cultured rat adipose tissue. *Biochem. J.*, **324**, 605–610.
[19] Halleux, C. M. *et al.* (1998). Multihormonal control of ob gene expression and leptin secretion from cultured human visceral adipose tissue: increased responsiveness to glucocorticoids in obesity. *J. Clin. Endocrinol. Metab.*, **83**, 902–910.
[20] Considine, R. V. *et al.* (1997). Dexamethasone stimulates leptin release from human adipocytes: Unexpected inhibition by insulin. *J. Cell. B*, **65**, 254–258.
[21] Walsh, C. H. *et al.* (1976). Studies in whole body potassium and whole body nitrogen in newly diagnosed diabetes. *Quart. J. Med.*, **178**, 295–301.
[22] Tuominen, J. A. *et al.* (1997). Leptin synthesis is resistant to acute effects of insulin in insulin-dependent diabetes mellitus patients. *J. Clin. Endocrinol. Metab.*, **82**, 381–382.
[23] Schmitz, O. *et al.* (1997). Effects of hypersinsulinaemia and hypoglycaemia on circulating leptin levels in healthy lean males. *Diabetes and Metab.*, **23**, 80–83.
[24] Malmstrom, R. *et al.* (1996). Insulin increases plasma leptin concentrations in normal subjects and patients with NIDDM. *Diabetologia*, **39**, 993–996.
[25] Saad, M. F. *et al.* (1998). Physiological insulinemia acutely modulates plasma leptin. *Diabetes*, **47**, 544–549.
[26] D'Adamo, M. *et al.* (1998). Increased ob gene expression leads to elevated plasma leptin concentrations in patients with chronic primary hyperinsulinemia. *Diabetes*, **47**, 1625–1629.
[27] Kamoda, T. *et al.* (1998). Serum leptin and insulin concentrations in prepubertal lean, obese and insulin-dependent diabetes mellitus children. *Clin. Endocrinol.*, **49**, 385–389.
[28] Casabiell, X. *et al.* (2000). Dual effect of insulin on *in vitro* leptin secretion by adipose tissue. *Biochem. Biophys. Res. Comm.*, **276**, 477–482.
[29] Mick, G. J. *et al.* (2000). Inhibition of leptin secretion by insulin and metformin in cultured rat adipose tissue. *Biochem. Biophys. Acta.*, **1502**, 426–432.
[30] Hanaki, K., Becker, D. J. and Arslanian, S. A. (1999). Leptin before and after insulin therapy in children with new-onset type I diabetes. *J. Clin. Endocrinol. Metab.*, **84**, 1524–1526.
[31] Hathout, E. H. *et al.* (1999). Changes in plasma leptin during the treatment of diabetes ketoacidosis. *J. Clin. Endocrinol. Metab.*, **84**, 4545–4548.
[32] Atta, N. *et al.* (1999). Changes in free insulin-like growth factor-1 and leptin concentrations during acute metabolic decompensation in insulin withdrawn patients with Type 1 diabetes. *J. Clin. Endocrinol. Metab.*, **84**, 2324–2328.
[33] Becker, D. J. and Weber, B. (1995). Pathophysiology of Diabetes Mellitus. Third edn. Clinical Paediatric endocrinology, Ed. Brook, C. G. D., Osney Mead, Oxford: Blackwell Science Ltd.
[34] Houseknecht, K. L. *et al.* (1996). Evidence for leptin binding to proteins in serum of rodents and humans: modulation with obesity. *Diabetes*, **45**, 1638–1643.
[35] Hill, R. A. *et al.* (1998). Leptin: its pharmacokinetics and tissue distribution. *Int. J. Obesity*, **22**, 765–770.
[36] Sinha, M. K. *et al.*, Evidence of free and bound leptin in human circulation. Studies in lean and obese subjects and during short-term fasting. *J. Clin. Invest.*, **98**, 1277–1282.
[37] Garibotto, G. *et al.* (1998). Inter-organ leptin exchange in humans. *Biochem. Biophys. Res. Commun.*, **247**, 504–509.
[38] Landt, M. *et al.* (1998). Plasma leptin concentrations are only transiently increased in nephrectomized rats. *Am. J. Physiol.*, **275**, E495–E499.
[39] Campbell, F. M. *et al.* (1998). Interaction of free fatty acid with human leptin. *Biochem. Biophys. Res. Commun.*, **247**, 654–658.

